# Assessing the Medication Adherence App Marketplace From the Health Professional and Consumer Vantage Points

**DOI:** 10.2196/mhealth.6582

**Published:** 2017-04-19

**Authors:** Lindsey E Dayer, Rebecca Shilling, Madalyn Van Valkenburg, Bradley C Martin, Paul O Gubbins, Kristie Hadden, Seth Heldenbrand

**Affiliations:** ^1^ Department of Pharmacy Practice University of Arkansas for Medical Sciences College of Pharmacy Little Rock, AR United States; ^2^ University of Arkansas for Medical Sciences Little Rock, AR United States; ^3^ Division of Pharmaceutical Evaluation and Policy Department of Pharmacy Practice University of Arkansas for Medical Sciences College of Pharmacy Little Rock, AR United States; ^4^ Division of Pharmacy Practice and Administration University of Missouri-Kansas City School of Pharmacy Springfield, MO United States; ^5^ UAMS Regional Programs Center for Health Literacy University of Arkansas for Medical Sciences Little Rock, AR United States

**Keywords:** smartphone, apps, adherence, medication, health literacy

## Abstract

**Background:**

Nonadherence produces considerable health consequences and economic burden to patients and payers. One approach to improve medication nonadherence that has gained interest in recent years is the use of smartphone adherence apps. The development of smartphone adherence apps has increased rapidly since 2012; however, literature evaluating the clinical app and effectiveness of smartphone adherence apps to improve medication adherence is generally lacking.

**Objective:**

The aims of this study were to (1) provide an updated evaluation and comparison of medication adherence apps in the marketplace by assessing the features, functionality, and health literacy (HL) of the highest-ranking adherence apps and (2) indirectly measure the validity of our rating methodology by determining the relationship between our app evaluations and Web-based consumer ratings.

**Methods:**

Two independent reviewers assessed the features and functionality using a 4-domain rating tool of all adherence apps identified based on developer claims. The same reviewers downloaded and tested the 100 highest-ranking apps including an additional domain for assessment of HL. Pearson product correlations were estimated between the consumer ratings and our domain and total scores.

**Results:**

A total of 824 adherence apps were identified; of these, 645 unique apps were evaluated after applying exclusion criteria. The median initial score based on descriptions was 14 (max of 68; range 0-60). As a result, 100 of the highest-scoring unique apps underwent user testing. The median overall user-tested score was 31.5 (max of 73; range 0-60). The majority of the user tested the adherence apps that underwent user testing reported a consumer rating score in their respective online marketplace. The mean consumer rating was 3.93 (SD 0.84). The total user-tested score was positively correlated with consumer ratings (*r*=.1969, *P*=.04).

**Conclusions:**

More adherence apps are available in the Web-based marketplace, and the quality of these apps varies considerably. Consumer ratings are positively but weakly correlated with user-testing scores suggesting that our rating tool has some validity but that consumers and clinicians may assess adherence app quality differently.

## Introduction

### Background

Evolution in our health care system and technological advances in recent years have sparked renewed awareness of medication nonadherence; however, it continues to be a problem for our society [1]. Nonadherence produces considerable health consequences and economic burden to patients [2-5]. The repercussions of nonadherence may ultimately impede the appropriate management of common chronic diseases.

Unintentional nonadherence is a category of nonadherence that implies that the patient intends to take their medications, yet fail to do so for reasons such as forgetfulness or thoughtlessness [2]. Numerous strategies aimed at improving unintentional nonadherence have been studied. The use of smartphone medication adherence apps to improve unintentional nonadherence is one innovative approach that has recently gained interest [2,6]. Data suggest the use of or ownership of a smartphone continues to increase. According to the Pew Research Center’s Spring 2015 Global Attitudes Survey, an estimated 89% of the US population used the internet or reported owning a smartphone [7]. Estimates in 2015 also revealed that 62% of smartphone owners in the United States used their smartphone to find information about a health condition [8]. Adherence apps must be downloaded and installed using a cellular connection; however, many do not require active internet connections to access information or provide medication reminders. These adherence apps can also combine all of the user’s medication-specific material into one source to provide a more efficient way for individuals to participate in their disease management and care [2,6]. Given the proliferation in smartphone use, adherence apps represent a more accessible approach to address unintentional nonadherence in that they are easy to obtain and are available at all times to provide information to patients about their medications.

The development of smartphone adherence apps has increased sharply since 2012. In 2012, there were approximately 160 unique medication adherence apps available [2]. In 2014, the number of adherence apps ballooned to more than 400 [6]. This substantial increase in the adherence app marketplace reflects the high demand for this tool. Literature evaluating the clinical app and effectiveness of smartphone adherence apps to improve medication adherence is generally lacking. Study results indicating that medication reminder systems (eg, short message service [SMS] text messages) can increase medication adherence and may offer promise that adherence apps may also improve adherence [9-11]. A randomized trial conducted in Spain found that an app can improve self-reported adherence, but similar results in the United States have not yet been reported [12]. One study revealed that kidney transplant recipients were interested in using a smartphone app to remind them to take their medications, but they also reported perceived barriers to using these apps (eg, turning the phone off periodically, annoying alarms, an app that is not flexible to work with irregular schedules, and so on) [13]. Currently, a first of its kind randomized controlled trial designed to rigorously evaluate an app’s effect on blood pressure and medication adherence is underway [14]. Research also demonstrates lower health literacy (HL) is associated with decreased odds of participating in mobile health (mHealth) interventions for diabetes [15,16]. Although published data regarding efficacy, safety, or clinical outcomes for adherence apps are scarce [14,17], patients are using these apps and many groups of people have positive attitudes toward the use of this type of tool [18]; therefore, health care providers should be able to serve as a resource for patients to help identify an app that may best suit their needs with regard to its adherence features, functionality, and level of HL [2,6].

Previously, our group developed methods to compare adherence apps and identify those that offered a wide range of features that may be the most appropriate to recommend to patients [2,6]. From that work, a searchable online resource (medappfinder.com) was developed to help patients and providers identify and compare adherence apps based on usability, HL, and features that meet their individual needs [6]. The methods and analyses performed to develop these resources were standardized and represented a health care provider viewpoint. Thus, a limitation to these efforts is that patient viewpoints were lacking. Given the number of adherence apps and the rate at which the marketplace expands, directly incorporating patient viewpoints into these assessments is not feasible. However, most apps generally have consumer reviews posted in their online marketplaces. Such consumer reviews for adherence apps represent the viewpoint of the patient or their caregiver. Although these reviews have not been rigorously or systematically analyzed, such an analysis could indirectly provide the patients’ viewpoint of a given adherence app.

### Objective of the Study

The objective of this study was to (1) provide an updated evaluation and comparison of available medication adherence apps in the marketplace by assessing the features, functionality, and HL of the highest-ranking adherence apps across 5 domains and (2) indirectly measure the validity of our rating methodology by determining the relationship between our app evaluations and online consumer ratings. We hypothesized that consumer ratings and our ratings would be modestly correlated.

## Methods

### Marketplace Search

In June 2015, an additional Web-based app marketplace (the Microsoft Store [Windows]) and those included in prior analyses (iTunes [iOS], Google Play [Android], Blackberry App World) were analyzed to find all available adherence apps capable of producing medication reminders. The following keywords: alarm, alert, med(s), and pillbox were added to our existing search terms (ie, adherence, compliance, dosage, dose, drug, medication(s), pharm, pharmacy, pill(s), prescription, remind, reminder, Rx, script, take, therapy, treat, and treatment) [2,6] and used to identify these apps. In addition, “health and diet,” “health care services,” and “medical guides” categories were used to identify any additional adherence apps that may have not been found using keyword searches. Adherence apps were cataloged using the full name, manufacturer, and operating system (OS). To be included in the analysis, adherence apps had to be available in English and claim to send medication reminders. For adherence apps with both a free or lite and paid or pro version, only the paid or pro versions were recorded, evaluated, and scored for possible user testing, but it was noted if there was also a free version. Since our focus was to rate apps that could be used in a general ambulatory population, apps specific to one type of medication (eg, insulin) or a single disease state (eg, hypertension) were excluded. Apps that were specific to an independent pharmacy and/or required a specific prescription number or insurance type (eg, Humara Pharmacy and NexJ Health Coach) were also excluded from evaluation.

### Initial Evaluation and Scoring

Adherence apps were evaluated using previously described processes [2,6], which were developed based on author consensus before adherence app evaluation, and thus, reflects perspectives of the authors (eg, academicians, clinical practitioners, HL specialists, and student pharmacists). This rating system includes 28 features divided into 4 domains: Adherence Attributes, Medication Management, Connectivity, and General Features. It was found that 27 of the 28 features were nominally weighted as previously described from 1 to 3 (1, modest; 2, moderate; and 3, high), based on the authors’ views on its relevance and impact in its respective domain [2,6]. To reflect the growing marketplace, the scoring for the feature “Latest Revision Update” was modified from our prior methods. This feature is scored based on the frequency of continued app updates or support by developers. For this analysis, like before, 3 points were awarded if the app had been updated in the 6 months before the analysis, and 2 points were awarded if the app had been updated more than 6 months, but 12 or less months before our analysis. However, an app that had not been updated in more than a year previously received 1 point [6], but in this analysis, this was changed so that an app that had been updated 13-18 months before the review, received 1 point. An app that had not been updated in more than 18 months before this review, received a score of 0. No other scoring modifications were made. Using this rating system, two investigators independently analyzed the developers’ claims along with available screenshots of each adherence app in the online marketplace to determine whether an app possessed any of the 28 author-identified features to assign a score. The initial scores based on developers’ claims were used to identify the 100 highest ranked unique adherence apps. Those available on multiple platforms (eg, Medisafe, which is available on both iOS and Android), were evaluated separately to identify any differences between the platforms.

### User Testing

To compare the developers’ claims against the actual quality and functionality, the top 100 highest-ranking unique adherence apps underwent user testing by the same two evaluators using previously described methods [6]. Briefly, adherence apps that could not be installed or set up by at least one researcher due to inefficiencies or malfunctions with the app were excluded from further evaluation (eg, apps that continuously crashed, apps requiring an activation code, and inability set-up reminders despite contacting the app developer). To maintain a total of 100 unique user-tested adherence apps, those excluded from user testing for any reason were replaced with the next highest-ranking unique app based on initial score. Adherence apps available on both iOS and Android were rated for both platforms and given two scores, which were cross-verified and any score discrepancies resolved. The sum of the weighted scores determined an initial score, which was used to rank the adherence apps. Counting multiplatform adherence apps only once, the top 100 highest-ranking apps were then identified for user testing. Apps in the top 100 highest-ranking apps that had not been updated in the last 18 months before review were not retested. Aside from receiving a zero for the feature “Latest Revision Date,” these adherence apps received the same initial and user-tested scores as in our previous analysis [6]. Each adherence app was installed using a smartphone or tablet and tested for a minimum of 4 days and a maximum of 7 days using a standardized 6-drug regimen similar to prior analyses (ie, vitamin E, 400 IU once daily; diltiazem, 120 mg twice daily; simvastatin, 40 mg once daily at bedtime; azithromycin, 500 mg once daily for 3 days and then stop; and alendronate, 35 mg once weekly); however, instead of a prednisone taper regimen used in our prior analyses [2,6], the 6th drug used for the standardized regimen was methylnaltrexone, which should be consumed 12 mg every other day. Adherence apps unable to create reminders for a once weekly medication (ie, alendronate) were tested over 4 days, whereas those with this capability were tested over 7 days.

To assess the 28 possible author-identified adherence app features, the two evaluators used the same rating scale used in the initial evaluation with one update to the scoring process. In the initial evaluation, the feature, “Capability of Complex Medication Instructions,” was scored dichotomously as “yes” or “no” to reflect the presence or absence of this feature as discerned from the available developers’ descriptions and/or screenshots. However, during user testing, this feature was assessed using three possible rating options to reflect the actual performance and the degree to which an app could perform this feature (ie, create reminders for multiple medications and schedules that comprise complex medication instructions). The score given to each app for this feature was based on the number of reminders created for the more complex medication regimens (ie, azithromycin, alendronate, and methylnaltrexone). The maximum score of 3 was awarded when an app could create reminders for all of these medications. If an app was able to create reminders for only two, it received a score of 2. Adherence apps that could only create a reminder for one of these medications were assigned a score of 1. A score of 0 was assigned if an app was unable to create reminders for any of these medications. In these cases, it was decided that assigning a 0 for this feature was preferable to complete exclusion because these adherence apps are still available for potential patients to download. In retaining these adherence apps, this information can be shared with consumers to avoid the possible frustration had they downloaded based solely on developers’ claims and screenshots of apps in the marketplace.

In addition to the 28-feature author-identified rating system, 12 HL-focused attributes were evaluated and scored using a tool endorsed by the Institute of Medicine (IOM) [19] as previously described by Heldenbrand et al [6]. A separate domain was created and each attribute was scored using a Likert scale (1-5) with a maximum of 60 points. To prevent skewing the total user-tested scores, this domain’s scores were scaled with one point to be added for each 12-point increment in the HL domain score. The 12 feature’s scores were summed with a possible range of 12-60. For example, a HL score of 1-12 had 1 point added to the user-tested score, and a score of 13-24 had 2 points added.

The user-tested scores were calculated using the sum of the four original domains scores (max possible: 68) and the HL domain score (max possible: 5), creating a maximum user-tested score of 73. The user-tested scores, including those for the HL domain, were cross-verified by the two evaluators. Any adherence apps with scoring discrepancies between the two evaluators were reinstalled, and it was confirmed whether or not that adherence app possessed a particular feature. Following cross-verification, each app was given a final user-tested score which was then used to rerank the adherence apps.

To indirectly measure the patients’ viewpoint of adherence apps, available consumer ratings and the number of app downloads as of June-July 2015 were obtained from Google Play and iTunes. Pearson product-moment correlations were estimated between consumer ratings, user-testing total scores, and domain scores as a check of the validity of our user rating methodology. Using the obtained consumer ratings, we evaluated correlations between our ratings system based on manufacture claims and consumer ratings. We expected to find medium correlations between consumer ratings and our user domain scores. To classify correlations, we used the empirically based thresholds for correlations of small <0.20; medium (moderate)=0.20-0.30; and large >0.30 [20,21]. Finally, ordinary least square regression models were estimated using consumer ratings as the dependent variable and individual domain scores and individual items as independent variables.

## Results

### Initial Search

There were 824 adherence apps discovered across the 4 platforms including 378 iOS, 305 Android, 105 Windows, and 36 Blackberry. After applying exclusion criteria, 179 adherence apps were excluded (17 duplicates and 162 specific to a single medication and/or disease state), leaving 645 eligible for evaluation. Of the remaining adherence apps, 71% (461/649) were available on only one platform (208 iOS, 156 Android, 20 Blackberry, and 77 Windows) and 29% (184/634) were available on multiple platforms. Of the 645 adherence apps assessed based on developer claims, the median score was 14 with a mean of 15.5 (SD 9.4) and ranged from 0 to 60 (max possible: 68).

### User Testing

From the initial assessment based on developer claims, the top 100 highest-ranking unique adherence apps underwent user testing to assess features and HL resulting in a user-tested score. Due to the multiplatform adherence apps, a total of 144 apps were tested (75 iOS, 62 Android, and 7 Windows). Blackberry adherence apps were excluded due to the lack of devices available to test these apps. During user testing, 34 adherence apps were unable to be installed and/or set up by at least one researcher and were ultimately excluded from further evaluation. The most common issues included apps that continuously crashed despite using multiple devices (eg, iPhone 5, iPhone 6s, and iPad for iOS apps), the need for a specific activation code from a primary care physician and/or pharmacy to set up and use the adherence app, and inability to set up reminders despite contacting the app developer which excluded 5, 11, and 8 apps, respectively. These were replaced by the next 34 highest-ranking adherence apps so that ultimately 100 unique apps were tested and given a user-tested score. The median overall user-tested score was 31.5 and ranged from 0 to 60 (max possible: 73). When compared with initial scores, 32% (46/144) of app scores decreased and 68% (98/144) of scores increased following user testing. Select features from each domain along with their frequencies can be found in Table 1. The overall user-tested score for the domains listed below can be found in Table 2. Table 3 reports the attribute scores of the 13 highest ranked adherence apps.

**Table 1:** Select adherence app feature frequencies based on user testing (max N=144).

Domain	Feature^a^	Frequency n (%)
Adherence Attributes	Customizable reminders	57 (39.6)
	Refill alerts	67 (46.5)
	Ability to postpone reminder	71 (49.9)
	Specific medication reminders	94 (65.3)
	Medical social networking	18 (12.5)
	Tracks missed or taken doses	88 (61.1)
Medication Management	Medication database	70 (48.6)
	Multiple profiles	69 (46.9)
	Food and drug interactions	15 (10.4)
	Refill from a specific pharmacy	14 (9.7)
Connectivity	Export or share information	93 (64.6)
	Reminders with no connectivity	82 (56.9)
	Cloud storage	81 (56.3)
	Patient Web portal	36 (25)
	Provider Web portal	15 (10.4)
General Features	HIPAA^b^ statement	15 (10.4)
	Multiplatform	90 (62.5)
	Multiple language options	30 (20.8)
	Completely free	87 (60)
	Password option	90 (62.5)

^a^Not a comprehensive list of features assessed.

^b^HIPAA: Health Insurance Portability and Accountability Act.

**Table 2:** Overall user-tested domain scores.

Domain	Median (range)	Max possible
Adherence Attributes	8 (0-21)	21
Medication Management	5 (0-14)	15
Connectivity	5 (0-10)	13
General Features	9 (1-16)	19
Health Literacy (scaled score)	36 (22-49.5) 4 (2-5)	60 (5)

**Table 3:** Highest-ranking adherence apps’ user-tested domain scores.

Apps	Operating system			Initial score	User-tested score	Adherence attributes	Medication management	Connectivity	General features	Health literacy	User-tested score+HL^a^	Latest revision date^b^
	iOS^c^	A^d^	W^e^	Max: 68	Max: 68	Max: 21	Max: 15	Max: 13	Max: 19	Max: 5	Max: 73	
Medisafe	X	X		47	55	21	10	8	16	5	60	July 2015
Care4Today	X	X		35	49	17	8	10	14	4	53	May 2015
CareZone	X	X		37	47	17	8	10	12	4	51	July 2015
MedPal		X		32	46	21	7	8	10	4	50	December 2013
MyMeds	X	X		38	47	18	9	10	10	3	50	March 2015
CareSync	X	X		30	44	12	10	10	12	4	48	June 2015
Dosecast Premium	X	X		43	44	16	8	8	12	3	47	July 2015
Mango Health	X	X		35	38	14	7	6	11	4	42	May 2015
GenieMD	X	X		35	38	8	13	7	10	3	41	January 2015
MyHealthSaverz	X	X		38	37	13	8	5	11	4	41	March 2015
Pill Reminder by Drugs.com	X			41	38	10	11	7	10	3	41	December 2014
eMedsMate			X	33	36	9	10	8	9	4	40	July 2014
ZibdyHealth		X	X	59	36	5	11	7	15	2	40	June 2015

^a^HL: health literacy.

^b^At the time apps were cataloged (June-July 2015).

^c^iOS: Apple.

^d^A: Android.

^e^W: Windows.

### Adherence Attributes

During user testing, 9% (13/144) of adherence apps were unable to reliably send medication reminders although the developers’ descriptions and/or available screenshots claimed to have this function, and thus received a 0 for their final user-tested score in this domain.

#### Medication Management

The ability of adherence apps to accurately send reminders on complex medication regimens varied greatly. It was found that 40% (58/144) of adherence apps were able to create reminders for all three medications with complex schedules (ie, azithromycin, alendronate, and methylnaltrexone) and 9% (13/144) were unable to create reminders for any of these medications.

#### Connectivity

**Figure 1:**
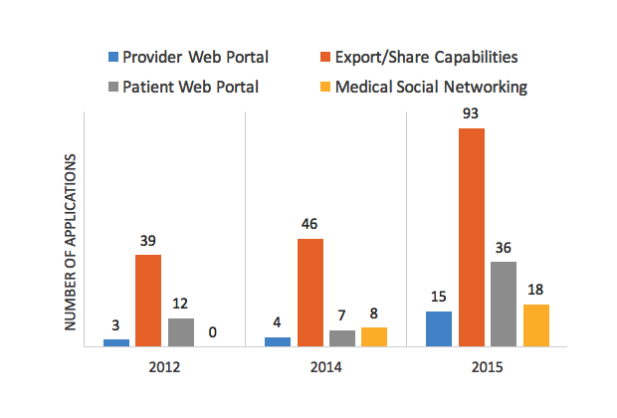
Changes in advanced connectivity features.

This domain has changed the most since previous medication adherence app studies [2,6] as seen in Figure 1. The number of adherence apps that can export or share information has grown 238% since initial analysis in 2012 (93 vs 39 apps, respectively). Medical social networking, a feature that allows the patient to choose caregivers or providers to actively monitor and participate in the patient’s medication adherence, was absent in all apps in 2012 [2], but was present in 13% (18/139) of adherence apps in this analysis.

#### General Features

Of the 57 adherence apps that had a pro or paid version, 60% (34/57) also had a free version. In total, 60% (87/144) were completely free. Adherence app prices ranged from free to US $23.99/year, with the average cost being a one-time purchase fee of US $1. Additionally, some adherence apps were free to download, but required in-app purchases to unlock extra features.

#### Health Literacy

The plain language feature had a median score of 1, which was the lowest-scoring feature of this domain. Adherence apps were given this score based on the number of words with greater than three syllables (eg, medication, prescription, and interaction). The highest-scoring features were the use of bullets and/or short lists and having adequate white space or little clutter, which both received a median score of 4.

### Relationship Between Adherence App Evaluations and Consumer Rating

Of the 645 adherence apps initially evaluated using developer claims, 67% (417/645) had a consumer rating score. The mean consumer rating was 3.54 (SD 1.14) with a minimum rating of 1 and a maximum rating of 5. The total score based on developers’ claims was positively and moderately related to consumer ratings (Table 4; *r*=.2399, *P*<.001). All domains were significantly positively correlated with consumer ratings, but only the General Features domain had a correlation of at least 0.20.

The majority of the 144 adherence apps that underwent user testing had a consumer rating score. The mean consumer rating was 3.93 (SD 0.84). The total user-tested score was positively and almost moderately correlated with consumer ratings (Table 4; *r*=.1969, *P*=.04). Only the Connectivity domain score was significantly and moderately correlated with consumer ratings, whereas the small correlation with the General Features domain score trended toward significance (*P*=.05). The HL domain score was significant and negatively correlated with consumer ratings (*r*=−.1942, *P*=.04).

Because the General Features domain based on user testing and developer claims were positively related to consumer reviews, ordinary least square regressions were estimated between the individual items within the General Features domain and the other domain total scores. The model explained 33% of the variability in consumer ratings of the adherence apps user tested (data not shown). Only one item of the General Features domain, whether the app was free or not, was significantly and positively related to consumer rating (beta=.43, *P*<.001).

**Table 4:** Pearson correlation coefficients between consumer ratings and adherence app evaluations based on developers claims and user testing.

Domains	Developers claims N=417	User testing N=144
Adherence Attributes	0.1631 (*P*<.001)	0.0569 (*P*=.55)
Medication Management	0.1302 (*P*<.001)	0.1488 (*P*=.12)
Connectivity	0.1275 (*P*<.001)	0.2362 (*P*=.01)
General Features	0.2575 (*P*<.001)	0.1845 (*P*=.05)
Health Literacy	N/A^a^	−0.1942 (*P*=.04)
Total score	0.2399 (*P*<.001)	0.1969 (*P*=.04)

^a^N/A: not applicable.

## Discussion

### Principal Findings

**Figure 2:**
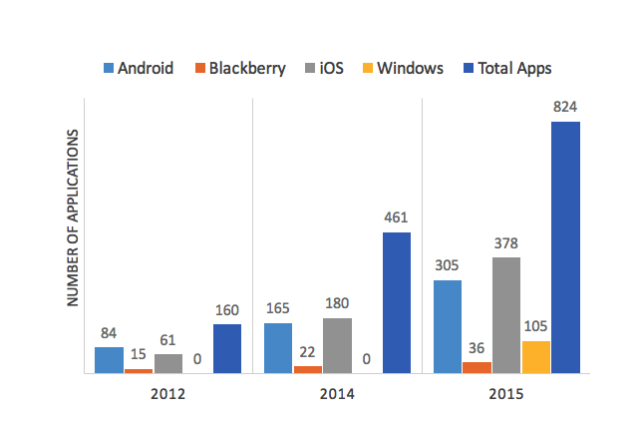
Changes in the online marketplaces.

There has been a 515% increase in the number of recorded adherence apps since 2012 (160 in 2012 vs 824 in 2015; Figure 2) [2]. The Windows platform, which was added to our analysis in 2015, had 105 adherence apps. This search revealed that 83 of the adherence apps recorded from the summer of 2014 could not be found in 2015. There continues to be extreme variability in the quality and functionality of adherence apps which could decrease the likelihood of consumers finding the best adherence app on their first attempt. Thus, resources that compare and contrast adherence apps for patients and providers (ie, medappfinder.com) could help in determining specific, desired features and help locate the highest-ranking adherence apps containing those selected features (Multimedia Appendices 1-3). Providers should be familiar with several quality adherence apps to recommend to patients as an additional tool for patients to use in addressing medication nonadherence.

Data on the effectiveness of adherence app medication reminder systems for a variety of disease states or specific populations are sparse, and the overall effectiveness of adherence apps for patients with chronic conditions is still unknown [12,22-26]. Therefore, there is a continued need for randomized controlled trials using rigorous research methodologies to establish an evidence-base for effective medication adherence apps.

The positive small to modest correlations between consumer ratings and our methods used to assess adherence apps provide some evidence of the validity of our approach in evaluating adherence apps. We expected to find modest correlations between our ratings and consumer ratings and these were observed empirically in our assessment based on developer’s claims and were almost of moderate strength for (*r*=.1969) user testing total scores. Not surprisingly, the General Features domain which assesses the frequency of updates, charges, advertisements, and photos was most strongly correlated with consumer reviews and the strongest individual item associated with consumer reviews was whether or not the adherence app was free of charge. Because there were many more apps with initial scores based on claims (n=417) compared with those with user-testing scores (n=144), the correlations for the user-tested scores bordered on significance while even small correlations for initial scores based on claims were significant. For some of the domain scores, we only found small correlations (*r*<.20), which was lower than anticipated. This may be due to low reliability of consumer ratings described below, possible reliability or validity issues with our approach in assessing the domains, or fundamental differences in how consumers and clinician perceive medication app quality.

However, the small yet significant negative correlation between our HL assessment and consumer ratings suggests our assessment of HL may need refinement. This disparity requires further investigation. It is possible that the consumer ratings may be measured with error, or perhaps the findings reflect a selection bias of persons who download and take the time to rate and write reviews of adherence apps. Such individuals by virtue of owning a smartphone and downloading the adherence app may have higher educational attainment and higher levels of HL. Consequently, they may be more likely to prefer more sophisticated language and displays than those recommended by IOM best practices, which address the needs of individuals with low HL. In our approach to assessing HL aspects of adherence apps, we adapted the IOM’s recommendations for designing health literate mobile apps into a scored scale that was used to evaluate each user-tested adherence app [19]. The scale was applied by two independent evaluators to assess HL and to increase the reliability of our approach. Such selection bias, if present, would result in poorer consumer ratings for adherence apps with simpler designs and features. Alternatively, the consumer rating scores may also not be reliably and validly recorded. Consumer reviews are the mean scores of individual reviews and some apps are reviewed by nearly 100,000 persons and others by less than 5 persons. Further complicating the interpretation of consumer reviews, Android adherence apps include reviews from all app versions, and thus, the reviews incorporate assessments of earlier app versions that may have since been updated and improved. Conversely, iOS adherence app reviews can be limited to the most recent version of the product. To mitigate this discrepancy between platforms, investigators recorded reviews from the iTunes marketplace using the “all versions” option. Considering that updating an adherence app does not guarantee an improvement in the overall product, these differences in reporting of consumer reviews in Web-based marketplaces complicates the analysis.

The top three highest-ranking adherence apps were updated within 1-3 months of data collection. This suggests how these adherence apps (ie, Medisafe, Care4Today, and CareZone) have kept their services well-run and efficient. These apps are also available on multiple OS’s which adds to their convenience. A new OS, Windows, was included and analyzed for this study and two adherence apps with this system were highly ranked initially (ie, eMedsMate and ZibdyHealth); however, after user testing, ZibdyHealth’s score dropped due to their Adherence Attributes domain which demonstrates the importance of the user testing portion of this project.

Recent advancement in adherence app connectivity features has increased 238% since our initial evaluation of the app marketplace in 2012 [2]. Medical social networking and the real-time adherence monitoring for both patients and providers are the two features in which notable advancement has been observed since our initial work, particularly among the more advanced adherence apps. Medical social networking allows the patient to choose caregivers or providers to be in their network and receive notifications if the patient misses a dose of their medication. This is especially helpful for the forgetful patient. Real-time adherence monitoring can generate encouraging adherence reports for patients, or be used by providers when assessing the patient’s response to medications during clinic visits. Other adherence app technologies that could be promising involve increasing interactivity of the app by offering rewards for desired behaviors with the ultimate goal of promoting habit formation [23,27,28]. This “gamification” and rewards strategy has been used successfully by apps in the mHealth sector for fitness, smoking cessation, and adolescent diabetes self-management [23,24,27,29-32]. Of course, these improvements in connectivity may improve user satisfaction or the value they place on the adherence app, but demonstrating that these advancements in functionality specifically improve medication adherence or patient outcomes will take considerable study.

### Limitations

The following limitations should be considered when interpreting the findings presented herein. As briefly discussed above, consumer ratings introduce potential selection bias, specifically when evaluating how “health-literate” an app is. Such potential is possible, because the demographics and other characteristics of consumers who rate and review adherence apps are unknown. Thus, it is not known if the raters are representative of users who may need adherence apps the most, namely those with poor health outcomes, often related to low HL. Second, the IOM recommendations for designing HL into mobile apps, which were used as rating criteria in this project to assess how “health-literate” the adherence apps are, were only promulgated in 2014. Therefore, it is unknown whether developers are fully aware of these recommendations or the extent to which they have purposefully tried to implement them into their adherence app designs. Although our rating system assesses the inclusion of features consistent with the IOM recommendations, it does not capture when these features were added or how well they address the IOM recommendations. Finally, the attributes and scoring approach reflect the health care provider and research perspectives and not necessarily the consumer or patient perspective. Our ratings, however, were positively correlated with consumer reviews suggesting that our ratings, at least in part, may also be useful to end users.

### Conclusions

Medication nonadherence, particularly unintentional nonadherence, continues to complicate the management of those with chronic illnesses. With ongoing proliferation of smartphones and mobile technology, the adherence app marketplace continues to rapidly expand. Past analyses of desirable attributes of adherence apps from a health care provider vantage point correlate with unsolicited consumer reviews of the apps. Although consumer-provided reviews of adherence apps provide an indirect method to assess the patient or caregiver perspective of adherence apps, they do not correlate with standardized health care provider assessments of the “HL” of such apps. Future studies are needed to improve the external validity of the HL assessments of adherence apps.

## Appendix

Multimedia Appendix 1 Medappfinder.com homepage.

Multimedia Appendix 2 Medappfinder.com patient app search page.

Multimedia Appendix 3 Medappfinder.com provider app search page.

## Abbreviations

HL: health literacy
IOM: Institute of Medicine
OS: operating system
SMS: short message service
