# Supplementary figures and images for: Assessing the Medication Adherence App Marketplace From the Health Professional and Consumer Vantage Points
Source: JMIR Mhealth Uhealth. 2017 Apr 19;5(4):e45. doi: 10.2196/mhealth.6582 (PMC5415657; doi:10.2196/mhealth.6582)

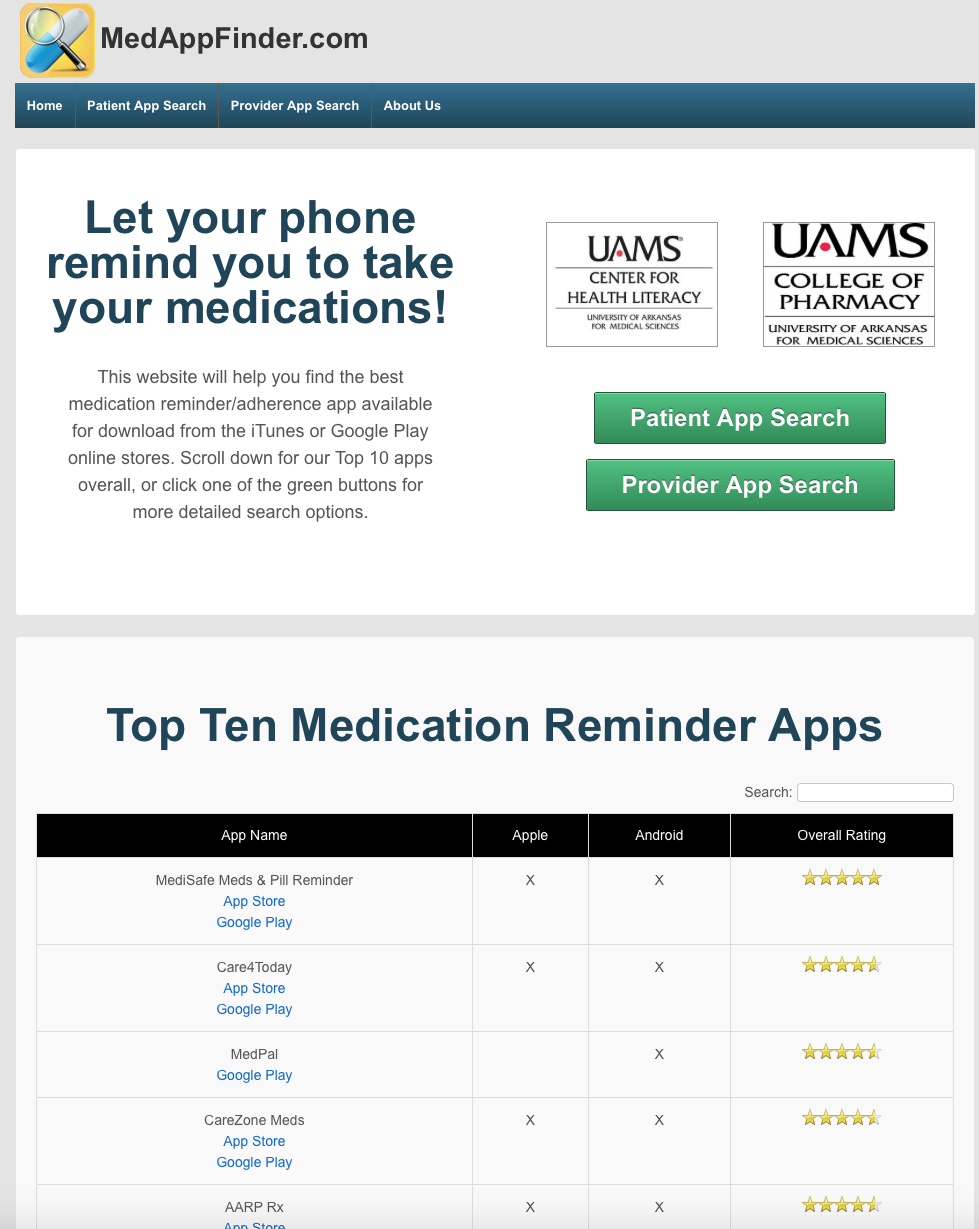

Supplement: Multimedia Appendix 1 [file mhealth_v5i4e45_app1.png]

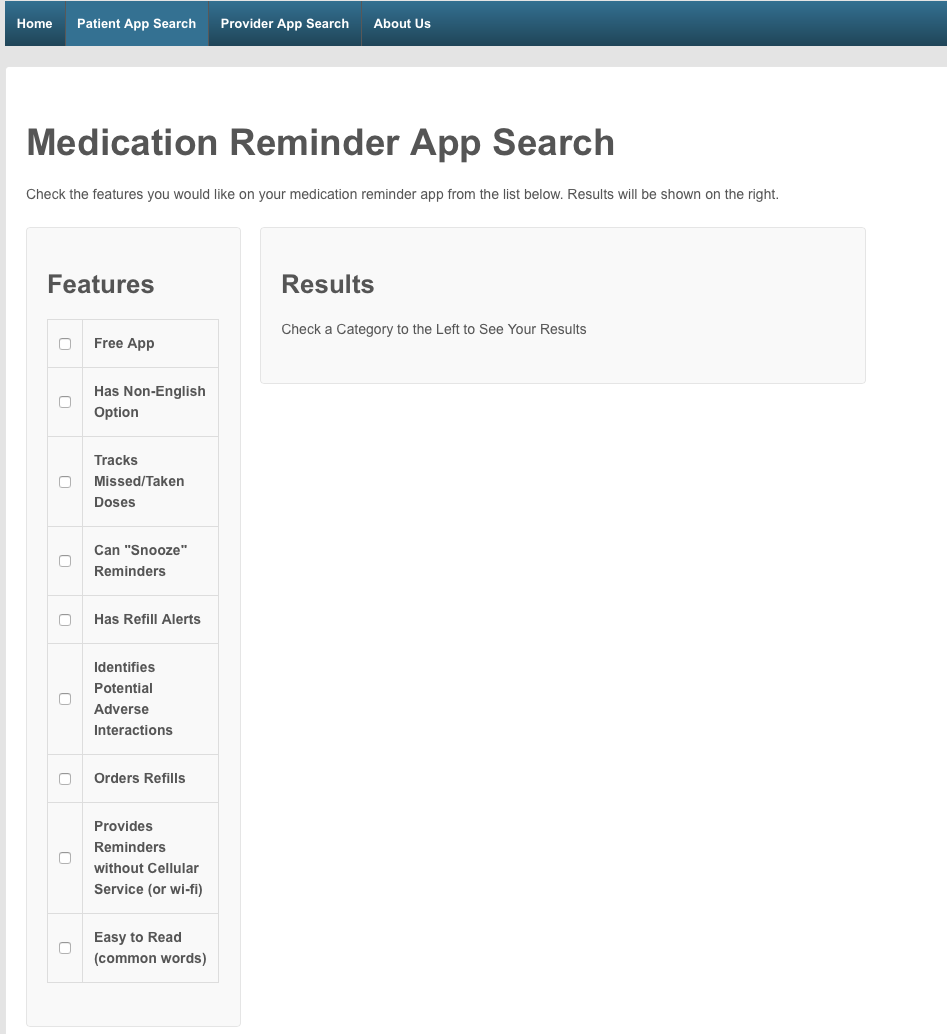

Supplement: Multimedia Appendix 2 [file mhealth_v5i4e45_app2.png]

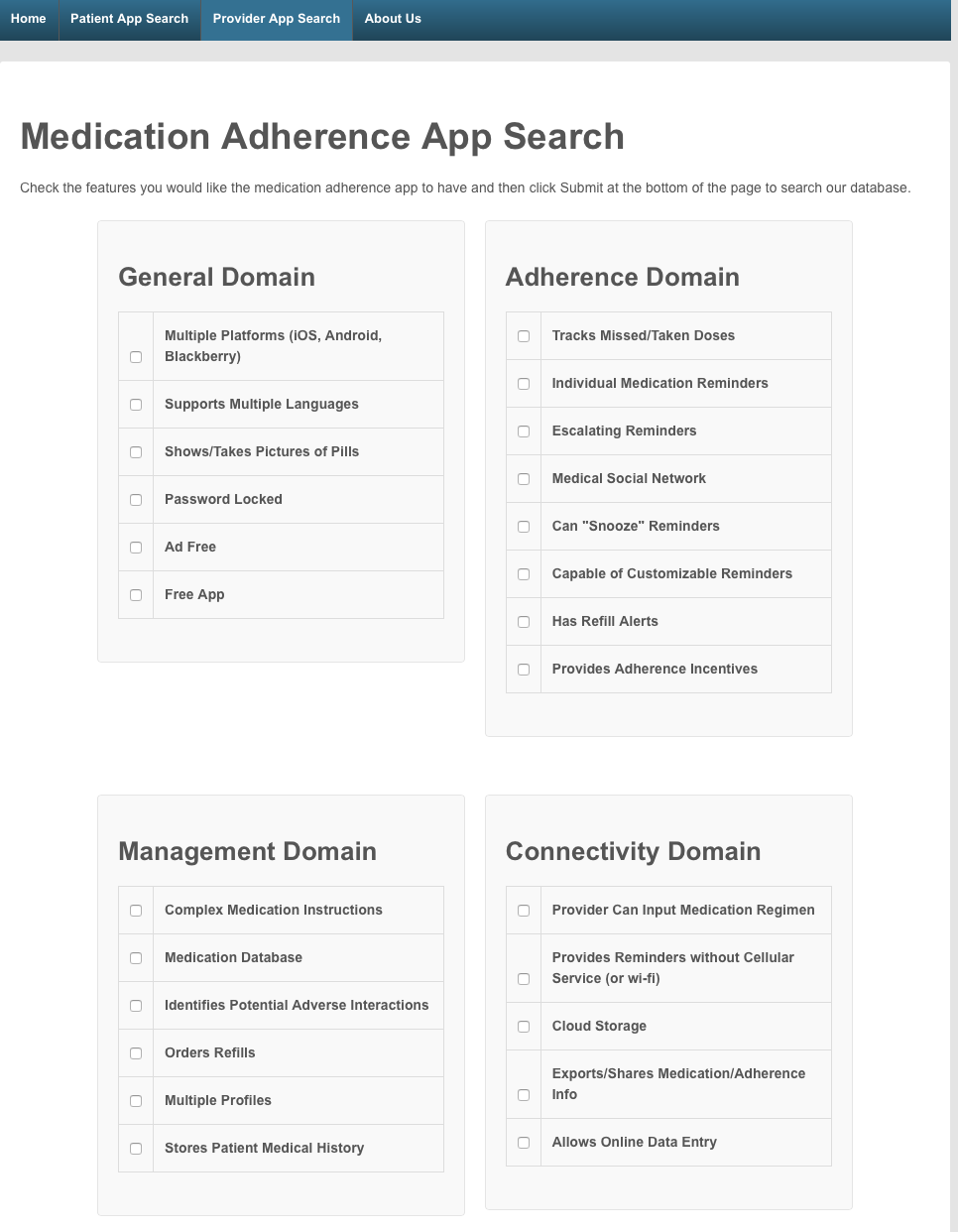

Supplement: Multimedia Appendix 3 [file mhealth_v5i4e45_app3.png]
